# Genome-wide detection of copy number variation in American mink using whole-genome sequencing

**DOI:** 10.1186/s12864-022-08874-1

**Published:** 2022-09-13

**Authors:** Pourya Davoudi, Duy Ngoc Do, Bruce Rathgeber, Stefanie M. Colombo, Mehdi Sargolzaei, Graham Plastow, Zhiquan Wang, Karim Karimi, Guoyu Hu, Shafagh Valipour, Younes Miar

**Affiliations:** 1 Department of Animal Science and Aquaculture, Dalhousie University, Truro, NS Canada; 2 Department of Pathobiology, University of Guelph, Guelph, ON Canada; 3 Select Sires Inc., Plain City, OH USA; 4 Livestock Gentec, Department of Agricultural, Food and Nutritional Science, University of Alberta, Edmonton, AB Canada

**Keywords:** American mink, Copy number variation, Whole-genome sequencing

## Abstract

**Background:**

Copy number variations (CNVs) represent a major source of genetic diversity and contribute to the phenotypic variation of economically important traits in livestock species. In this study, we report the first genome-wide CNV analysis of American mink using whole-genome sequence data from 100 individuals. The analyses were performed by three complementary software programs including CNVpytor, DELLY and Manta.

**Results:**

A total of 164,733 CNVs (144,517 deletions and 20,216 duplications) were identified representing 5378 CNV regions (CNVR) after merging overlapping CNVs, covering 47.3 Mb (1.9%) of the mink autosomal genome. Gene Ontology and KEGG pathway enrichment analyses of 1391 genes that overlapped CNVR revealed potential role of CNVs in a wide range of biological, molecular and cellular functions, e.g., pathways related to growth (regulation of actin cytoskeleton, and cAMP signaling pathways), behavior (axon guidance, circadian entrainment, and glutamatergic synapse), lipid metabolism (phospholipid binding, sphingolipid metabolism and regulation of lipolysis in adipocytes), and immune response (Wnt signaling, Fc receptor signaling, and GTPase regulator activity pathways). Furthermore, several CNVR-harbored genes associated with fur characteristics and development (*MYO5A*, *RAB27B*, *FGF12*, *SLC7A11*, *EXOC2*), and immune system processes (*SWAP70*, *FYN*, *ORAI1*, *TRPM2*, and *FOXO3*).

**Conclusions:**

This study presents the first genome-wide CNV map of American mink. We identified 5378 CNVR in the mink genome and investigated genes that overlapped with CNVR. The results suggest potential links with mink behaviour as well as their possible impact on fur quality and immune response. Overall, the results provide new resources for mink genome analysis, serving as a guideline for future investigations in which genomic structural variations are present.

**Supplementary Information:**

The online version contains supplementary material available at 10.1186/s12864-022-08874-1.

## Background

Copy number variations (CNVs), mainly refer to deletion or duplication of DNA segments, are a particular form of genomic structural variation ranging from 50 bp to several megabases (Mb) [1]. Although CNVs are less frequent compared to single nucleotide polymorphisms, due to their greater size, they might have large effects as a result of altering gene dosage, disrupting coding sequence and modifyng gene expression [2], leading to significant impacts on phenotypes of economic interest [3–5]. In addition, CNVs are associated with disease susceptibility [6–11], and might contribute to substantial part of missing heritability [12]. It was shown that CNVs play a critical role in regulating several complex diseases in human including autism [7], breast cancer [8], schizophrenia [9], depression [10], and susceptibility to Coronavirus [11]. Similarly, CNVs have been suggested to be responsible for traits and diseases in domesticated animals, such as polled intersex syndrome in goats [13], susceptibility to melanoma in horses [14], osteopetrosis in cattle [15], and dominant white color in pigs [16].

The decreasing costs of whole-genome sequencing (WGS) have made it feasible to map CNV with high resolution and accuracy [17]. Multiple approaches have been developed for WGS-based CNV detection, which use paired-end mapping, read-depth, and split-read [17]. The paired-end mapping method is applicable to paired-end reads and performs better in detection of CNVs in low-complexity regions [17]. On the other hand, the read-depth method relies on the depth of coverage in genomic regions and utilizes the changes in read depth to detect the CNV [18], and can identify large CNVs in complex genomic regions [19]. The split-read method refers to sequences that map to the reference genome only at one end, with other partially or unmapped reads providing the location of the breakpoint [17].

Characterisation of CNV has been widely studied in livestock species such as cattle [20–22], sheep [23–25], goat [26–28], pig [29–31], chicken [32–34], turkey [35, 36], buffalo [37], yak [38, 39], and rabbit [40], indicating that CNVs might have significant impacts on the economically important traits [41–44]. However, to our knowledge, there is no genome-wide CNV study in American mink. Therefore, the objectives of the current study were to: 1) provide the first large-scale CNV map in American mink using whole-genome sequence data; 2) define sets of high confidence CNV regions (CNVR) by incorporating multiple approaches; and 3) examine the potential impacts of CNVR and their overlapped genes on traits of economic interest for mink selection programs through in-depth functional annotation analyses.

## Methods

### Animals and sampling

All procedures applied in this study were approved by the Dalhousie University Animal Care and Use Committee (certification# 2018-009, and 2019-012), and mink used were cared for according to the Code of Practice for the Care and Handling of Farmed Mink guidelines [45]. The study is reported in compliance with the ARRIVE guidelines.

All individuals were raised through standard farming condition and were euthanized in December 2018 [46]. Tongue samples were collected from two different farms, the Canadian Center for Fur Animal Research (CCFAR) at Dalhousie Faculty of Agriculture (Truro, NS, Canada) and Millbank Fur Farm (Rockwood, ON, Canada). All mink from Millbank Fur Farm were Black in color (*n* = 15), and individuals from CCFAR varied in color types, including Demi (*n* = 32), Mahogany (*n* = 20), Black (*n* = 16), Pastel (*n* = 10), and Stardust (*n* = 7). To keep the relationship between individuals low, we checked the pedigree information and selected individuals with the lowest degree of kinship for the further analyses (median = 0.015; 1st–3rd quantile of relatedness = 0.008–0.039). More details were provided about the studied individuals by Karimi et al. [47].

### Quality control and read alignment

Using the DNeasy Blood and Tissue Kit (Qiagen, Hilden, Germany), we extracted genomic DNA from tongue tissue samples in accordance with the manufacturer’s protocol. Sequencing (100 bp pair-end reads) was performed by BGISEQ-500 platform at Beijing Genomics Institute (BGI, Guangdong, China). Low-quality reads and adapter sequences were removed by using the SOAPnuke software version 2.1.5 [48]. Then, high-quality reads were aligned to the latest American mink reference genome (https://www.ncbi.nlm.nih.gov/assembly/GCF_020171115.1/) using Burrows-Wheeler Aligner version 0.7.17 [49] with default parameters. The conversion of aligned files to binary alignment map (BAM) format and subsequent sorting was performed with SAMtools version 1.11 [50]. Duplicates were then removed using the MarkDuplicates command tool of Picard version 2.0.1 [51]. Finally, the BAM files were indexed by SAMtools software version 1.15 [50].

### Identification of CNV

To increase the accuracy of CNV detection, we employed three software programs, including CNVpytor version 1.2.1 [52], DELLY version 0.9.1 [53], and Manta 1.6.0 [54]. The CNVpytor software applies a read-depth approach, and both DELLY and Manta use paired-end and split-read methods. For each individual, the sorted BAM file was processed by CNVpytor [52], which is a Python version of its ancestor CNVnator [18]. Although both perform the same procedures, we applied CNVpytor as it is considerably faster in computational time [52]. The CNV calling was carried out by setting a bin size of 100 bp, following the recommendation of Abyzov et al. [18]. For improving the CNV detection accuracy, the following criteria were set to filter false positive candidates: the CNV calls with *P*-value < 0.01, sizes greater than 1 kb, fraction of mapped reads with zero quality (q0) > 50%, fraction of N bases (i.e., unassembled reference genome) within call region (pN) > 5%, and the distance to nearest gap in reference genome (dG) > 100,000. In the current study, we removed CNVs smaller than 1 kb to avoid noises, since most of the CNVs calling algorithms had low accuracy for small CNVs [17]. DELLY [53] and Manta [54] were performed with default parameters. The calls were filtered by removing the following 1) calls that were flagged IMPRECISE, 2) calls that did not pass the quality filters as suggested by DELLY and Manta (flag PASS), and 3) calls that had sizes smaller than 1 kb. Although DELLY and Manta had the ability to detect translocations and inversions events, we only considered deletions and duplications to have comparable results with the CNVpytor software. Only deletions and duplications were kept for further analyses. To generate a high-confident consensus call from different software, we implemented SURVIVOR version 1.0.3 [55] with default parameters, which merged the calls together with a maximum allowed distance of 1 kb, and CNVs with at least two out of three callers were kept for further analyses. This procedure cut down the false positive rate, yet without significantly reducing the sensitivity [55].

### Determination of CNVR

The CNVR were obtained by the CNVruler software version 1.2 [56], merging CNVs among individuals with at least 50% reciprocal overlap in their genomic coordinates. For instance, considering two CNVs, CNV1 starts at position X and ends at position Y, and CNV2 from Z to W, with X < Z < Y < W. Then if the reciprocal overlap between the two CNVs is at least 50%, the software merges them as a CNVR that runs from **X** to **W** on the genome [57]. To reduce the false positive rate, only the CNVR found in more than two samples were considered for further analyses [58]. The CNVR were categorized as gain or loss. The overlapping “loss” and “gain” CNVR were merged into single regions and called “mixed” CVNRs.

### Functional enrichment analysis of candidate genes overlapped with CNVR

A list of genes in the mink genome was downloaded from the NCBI website and Bedtools version 2.30.0 (function:intersect) [59] and was used to catalogue genes in corresponding regions. The Gene Ontology (GO), functional annotation and Kyoto Encyclopedia of Genes and Genomes (KEGG) pathway analyses [60] was carried out using the g:Profiler [61]. Analyses were performed using R packages including gprofiler2 version 0.2.1 [62], clusterProfiler version 3.0.4 [63], enrichplot version 1.16.1 [64], and org. Hs.eg.db version 2.7.1 [65]. All enrichment functions were selected through false discovery rate corrections and pathways with adjusted *P*-values < 0.05 were considered to be significant.

## Results

### Detection of CNVs

**Fig. 1:**
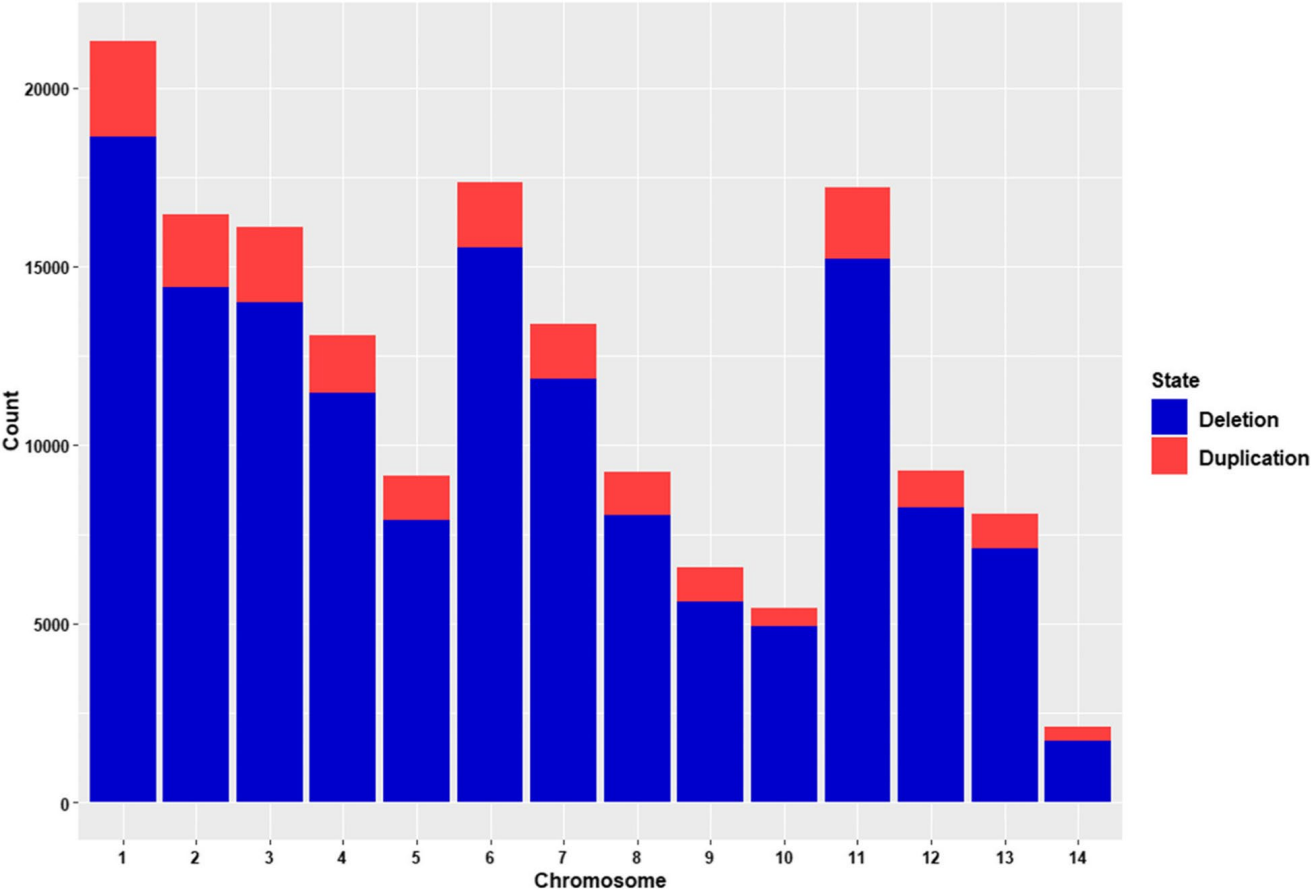
Numbers of CNVs identified across autosomal chromosomes of American mink

We employed different software including CNVpytor, DELLY, and Manta to detect CNVs in 100 American mink using WGS data. After merging the results of these methods, we retrieved a total of 164,733 CNV events (including 144,517 deletion and 20,216 duplication events) (Table 1), with an average number of 1647.3 per animal. The length size of identified CNVs ranged from 1 kb to 4255 kb with an average size of 7.4 kb. The detailed information of detected CNVs is provided in Additional file 1: Table S1. The CNVs were distributed over 14 autosomes with varying numbers in each autosome (Fig. 1).

**Table 1:** Descriptive statistics of CNVs detected in American mink genome

CNV			Length (bp)	
	Number	Mean	Minimum	Maximum
Deletion	144,517	6432.2	1000	3,171,151
Duplication	20,216	14,655.3	1003	4,254,987
Overall	164,733	7441.3	1000	4,254,987

### Number and distribution of CNVR

**Fig. 2:**
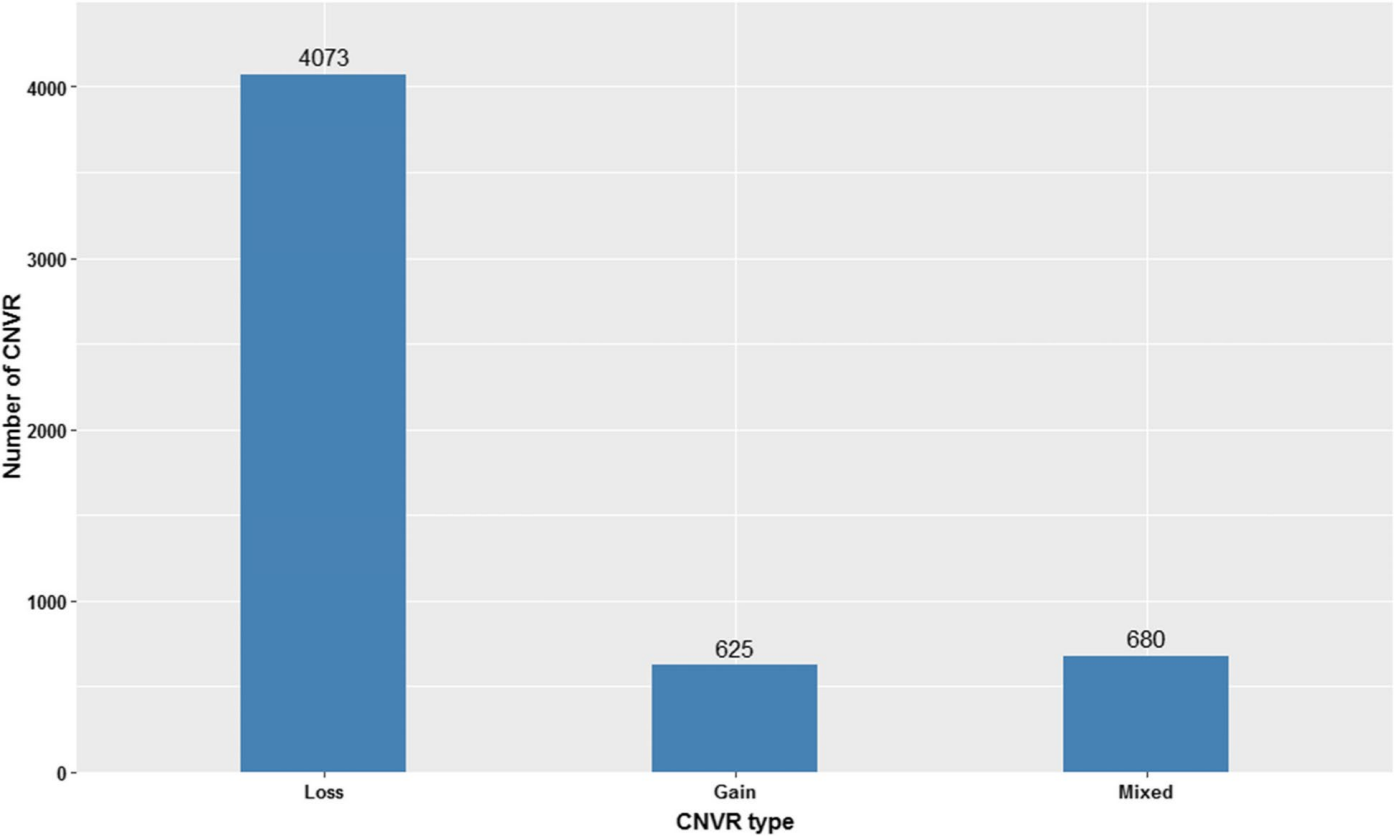
Distribution of CNVR types in American mink

A total of 5378 CNVR were obtained by merging overlapping CNVs across all individuals that covered 47.3 Mb of mink genome corresponding to 1.9% of autosomal genome sequence (Table 2). The CNVR included 4073 losses, 625 gains, and 680 mixed (loss and gain) events (Fig. 2). To achieve high-confident CNVR, we only considered CNVR identified in two or more samples. The size of CNVR varied from 1 to 3171.5 kb with an average of 8.9 kb. The largest number of CNVR were on chromosome 1 (683) and the lowest number were observed on chromosome 14 (82), which is in accordance with chromosome lengths.

**Table 2:** Distribution of CNVR across autsomal chromosmes of American mink genome

Chromosome	Chromosome length (bp)	CNVR count	Length of CNVR (bp)	Coverage (%)	Max size (bp)	Average (bp)	Min size (bp)
**1**	317,036,279	683	4,071,099	1.3	371,616	5960.6	1003
**2**	240,416,976	522	4,470,485	1.9	858,878	8564.1	1016
**3**	235,645,773	508	3,550,404	1.5	1,786,562	6988.9	1003
**4**	231,359,643	433	2,209,544	1	234,143	5102.9	1003
**5**	167,246,402	324	4,406,049	2.6	3,171,454	13,598.9	1019
**6**	224,559,537	543	2,456,160	1.1	150,398	4523.3	1004
**7**	207,076,058	417	2,699,685	1.3	664,002	6474.1	1012
**8**	144,012,018	273	2,135,038	1.4	955,355	7820.7	1009
**9**	101,698,841	224	1,068,011	1.1	229,614	4767.9	1004
**10**	75,573,270	189	2,509,561	3.3	1,866,663	13,278.1	1005
**11**	220,349,319	569	11,245,345	5.1	2,939,814	19,763.4	1003
**12**	148,690,698	319	1,804,339	1.2	652,086	5656.2	1003
**13**	152,771,447	292	4,030,656	2.6	1,986,383	13,803.7	1004
**14**	46,742,321	82	633,928	1.4	367,849	7730.9	1018
**Overall**	2,513,178,582	5378	47,290,304	1.9	3,171,454	8859.5	1003

**Fig. 3:**
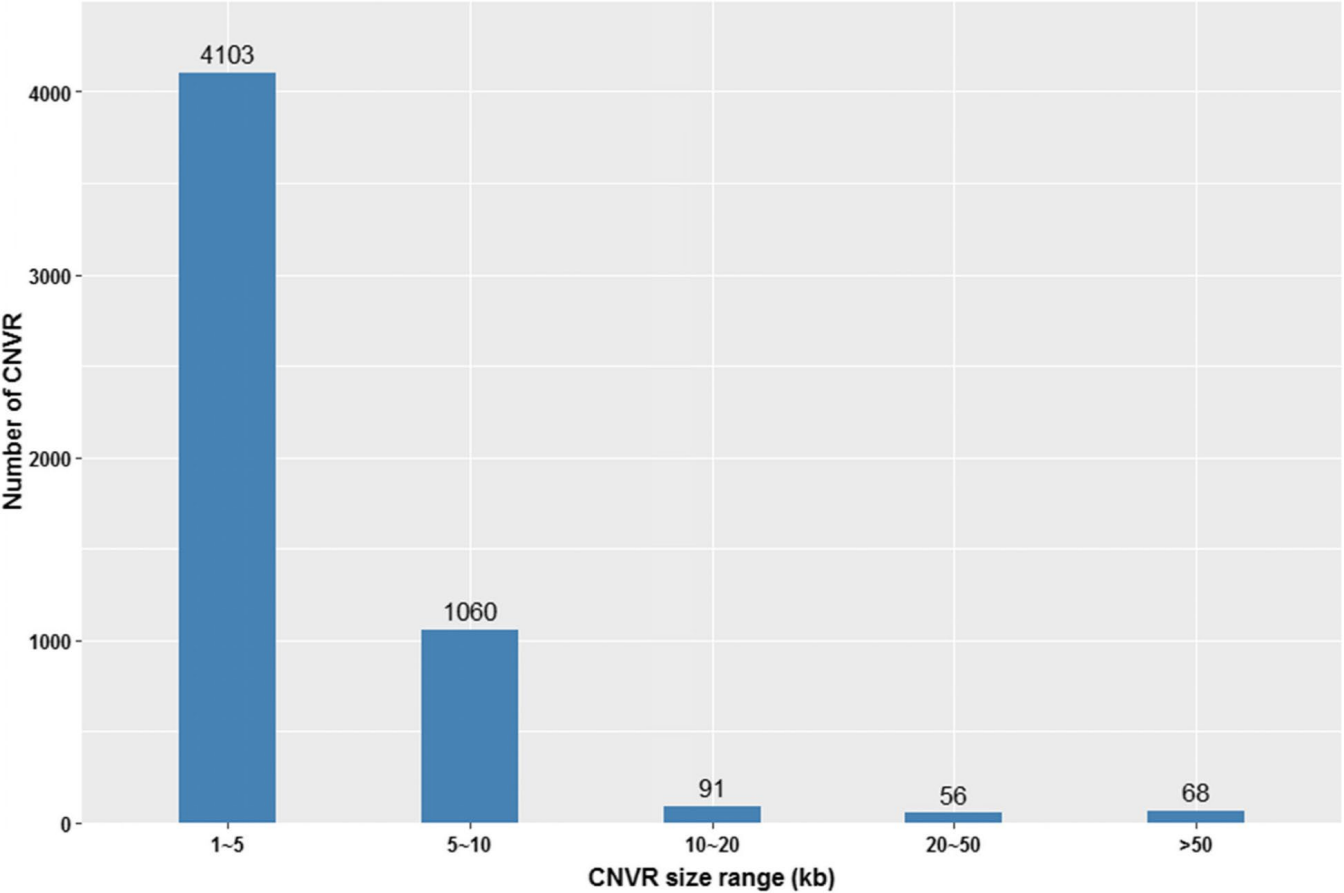
Distribution of CNVR sizes in American mink

In total, 4103 out of 5378 CNVR (76.3%) had sizes within 1–5 kb interval, following by 1060 (19.71%) within 5–10 kb, 91 (1.69%) within 10–20 kb, 56 (1.04%) within 20–50 kb, and 68 (1.26%) greater than 50 kb in length (Fig. 3).

**Fig. 4:**
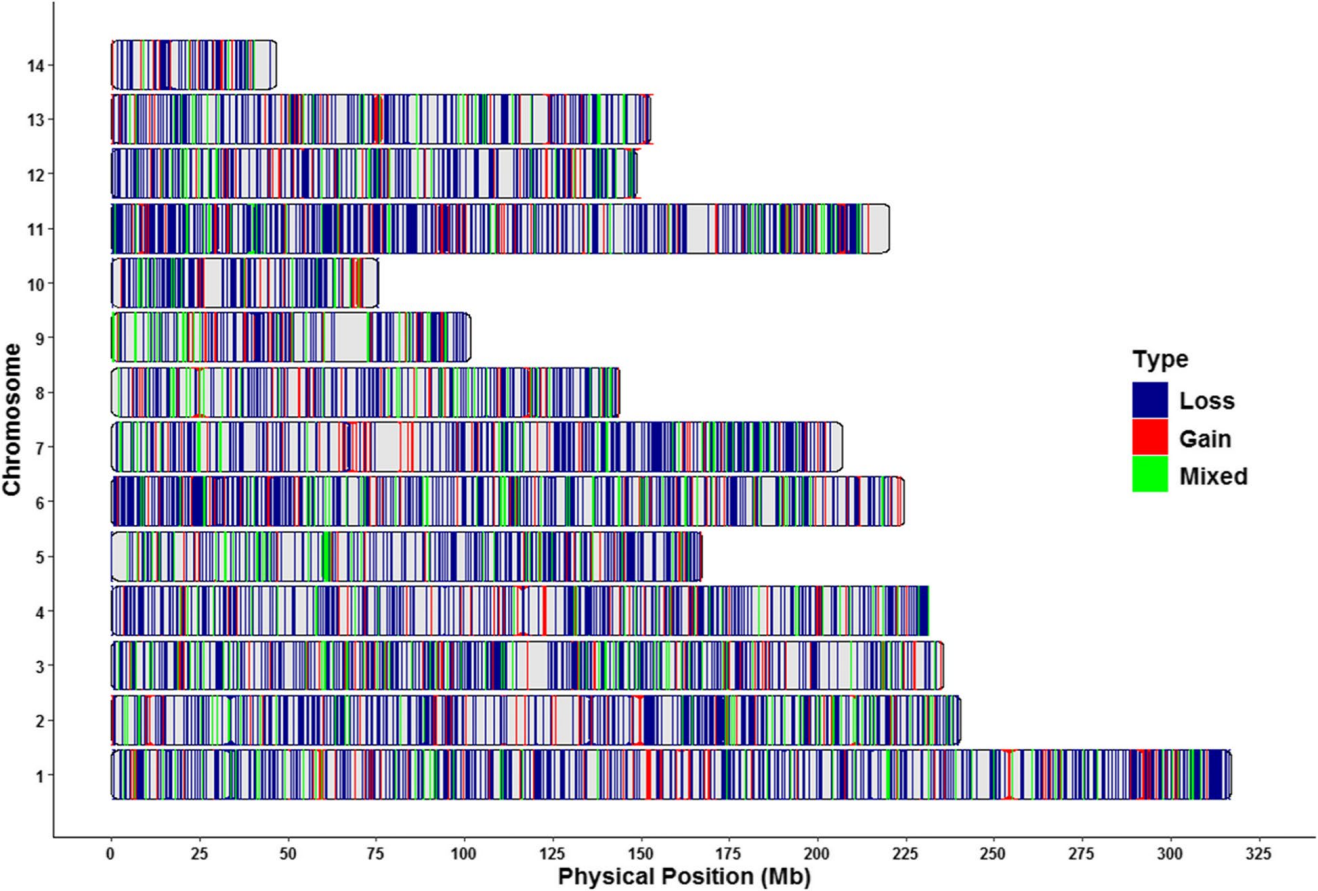
Genomic landscape of CNVR in American mink

The number of individuals supporting the CNVR varied from 2 to 98 out of 100 individuals, concentrating at 40.2% with 2-10 individuals, and only 5.6% of detected CNVR were observed in more than 90 individuals. The detailed information of all detected CNVR is provided in Additional file 1: Table S2. Furthermore, the physical locations of CNVR across the mink genome are presented in Fig. 4.

### Functional annotation and gene enrichment analyses

**Fig. 5:**
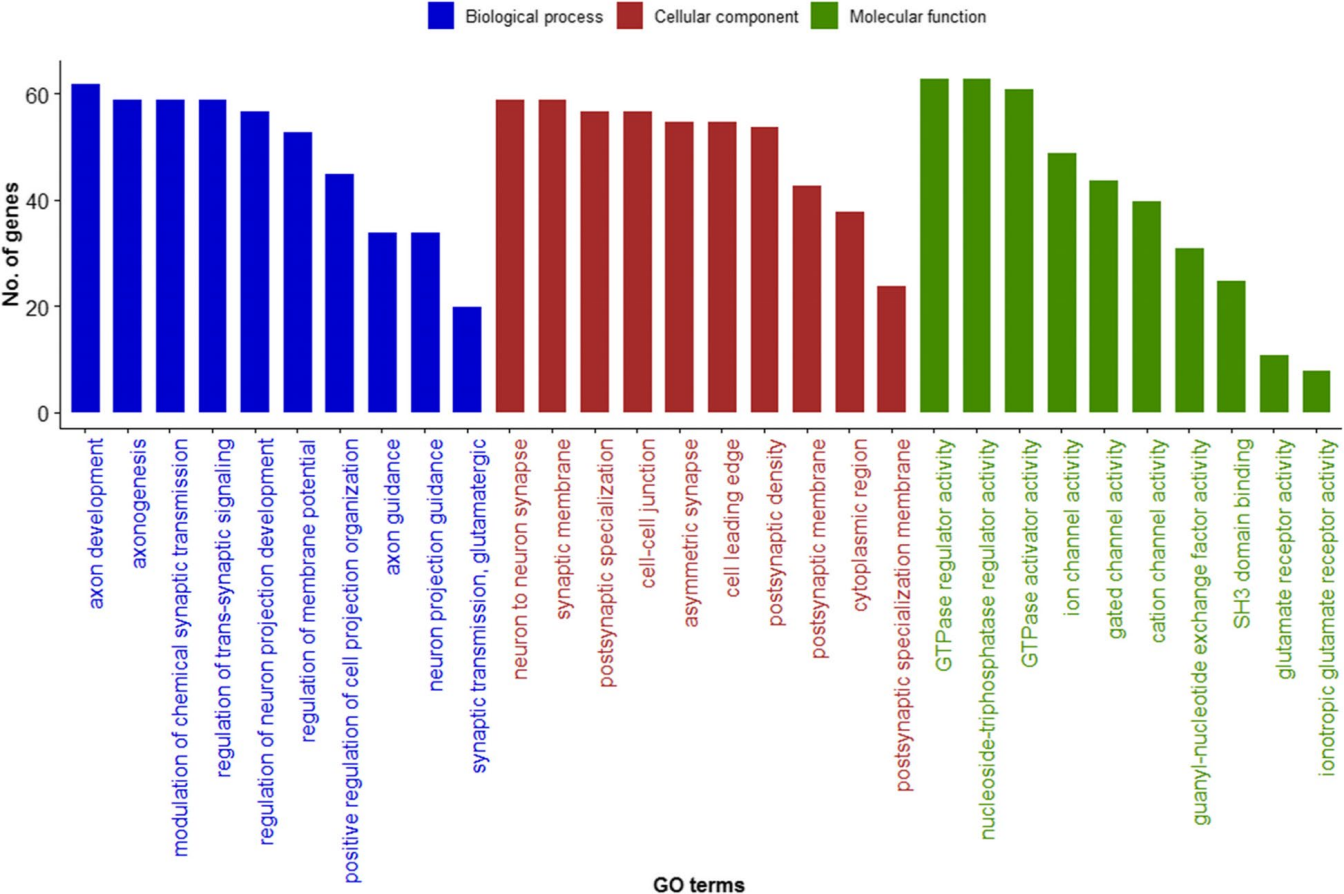
The top ten significant gene ontology terms enriched in CNVR-harbor genes

Analysis of the CNVR gene content revealed 1391 genes within or partially overlapped with 1878 (34.9%) detected CNVR (Additional file 1: Table S3). The enrichment analyses revealed 279 significant gene ontology (GO) terms (Additional file 1: Table S4) and 21 significant KEGG pathways (Additional file 1: Table S5). The results of GO analysis revealed that CNVR were significantly enriched (*P*-value < 0.05) in different biological functions e.g., axon guidance, phospholipid binding, Fc receptor signaling pathway, and GTPase regulator activity. The top ten significant GO terms enriched in CNVR-harbored genes were listed in the following GO categories (biological process, cellular component, molecular function) as depicted in Fig. 5.

**Fig. 6:**
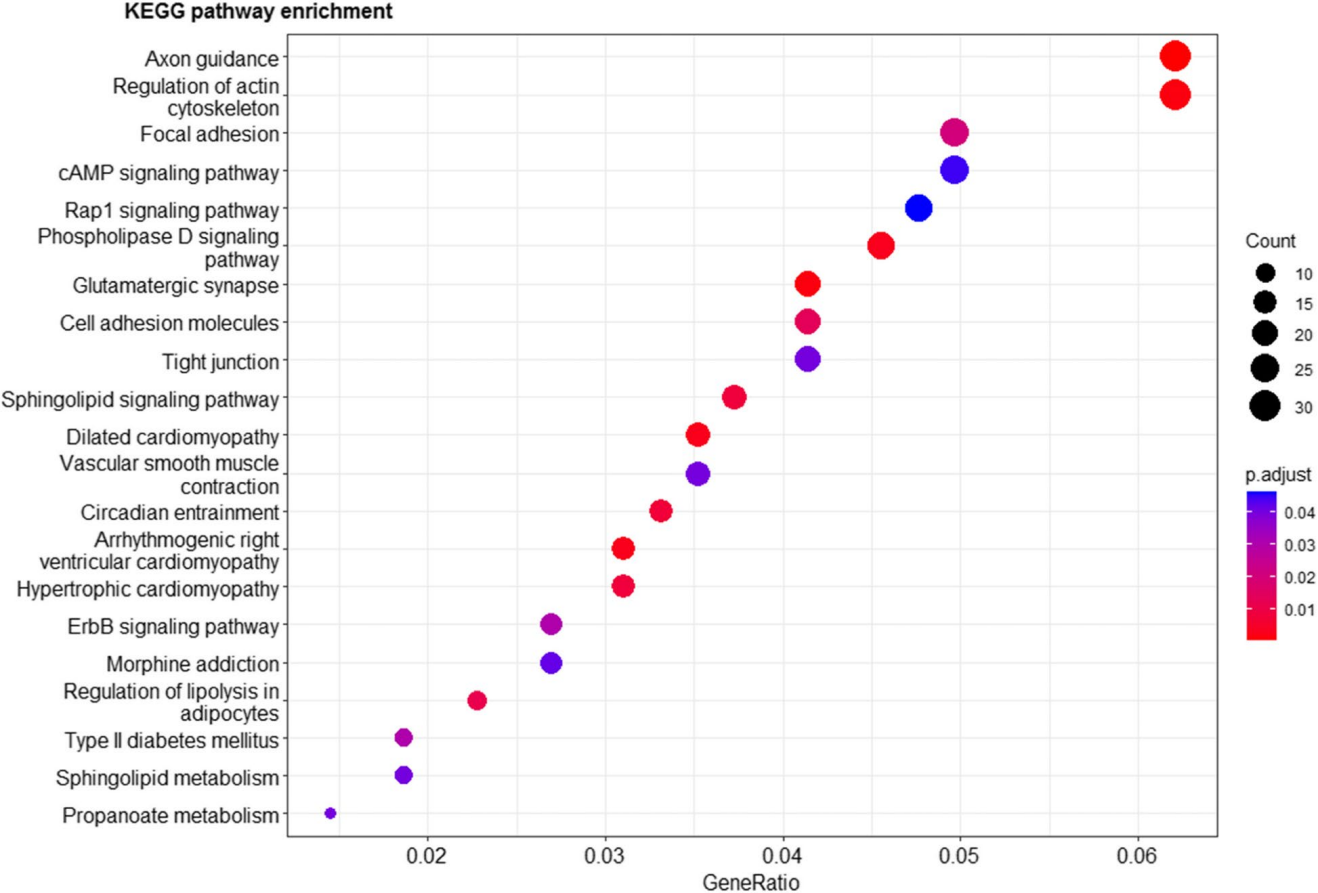
The KEGG pathways enriched in CNVR-harbor genes

In addition, the KEGG pathway analysis revealed 21 significantly enriched pathways (Fig. 6). These genes are mainly related to the axon guidance, glutamatergic synapse, regulation of actin cytoskeleton, cAMP signaling pathway, sphingolipid metabolism, and regulation of lipolysis in adipocytes (Fig. 6). The results of GO enrichment and KEGG analyses revealed the biological functions of several genes associated with fur characteristics and development (*MYO5A, RAB27B, FGF12, SLC7A11*, and *EXOC2*), and immune system processes (*SWAP70, FYN, ORAI1, TRPM2*, and *FOXO3*).

## Discussion

American mink (*Neogale vison*) is well-known as one of the most important sources of fur across the world [66]. It is essential for the mink industry to implement highly efficient breeding plans to meet sustainable production requirements [47]. Genome-wide identification of CNVs can provide new insights into genomic variations, which can assist in developing genomic breeding strategies for American mink. Numerous studies have been performed to identify CNVR in other species e.g., cattle [20], pig [43], goat [26], sheep [23], chicken [17], and buffalo [37]. Several studies indicated that CNVs could be highly associated with economically important traits in these species [29, 67–69]. To our knowledge, the current study provides the first genome-wide CNV detection in American mink.

We performed the CNV analyses on mink genome using WGS data. In total, we identified 164,733 CNV events (144,517 deletions and 20,216 duplications) with the average number of 1647.3 per mink. Similar results were reported in other livestock species e.g., dairy cattle (182,823 CNVs) [70], yak (98,441 CNVs) [39], Nellore cattle (195,873 CNVs) [71], and goat (208,649 CNVs) [26]. Some other studies reported a wide range of CNVs from 12 CNVs in chicken [72] to 1,747,604 CNVs in sheep [23]. This discrepancy might be due to the differences in the sample size, algorithms used for CNV calling, and sequencing technology [73]. A considerable number of detected CNVs were deletions (88.7%) in our study, which was expected because of the limited ability of the current algorithms in detection of insertions [74]. In addition, the detection of insertion is more diffucult in end mapping methods, since they only detect the duplications when mapped reads are shorter than the fragmented length [74].

The results showed that 5378 CNVR covered around 47.3 Mb (1.9%) of the mink genome, which falls within the range of several studies reported in other species, such as pig (1.72%) [75], cattle (2.5%) [42], chicken (1%) [32], quail (1.6-1.9%) [76], horse (1.3%) [77], and buffalo (2%) [37]. The CNVR covered the genome in different ranges in other species, including cat (0.3%) [78], pig (0.9%) [57], yak (6.2%) [38], [78] goat (10.8%) [26], chicken (12.8%) [69], and cattle (13%) [71]. Several reasons might affect the quantity of CNVR detection such as the detection algorithm, population size, genetic background, the quality of applied technology, and the differences in genome size [73, 79].

The results showed that 1391 genes in the mink genome were harbored within the detected CNVRs (34.9% of the total detected CNVRs). The GO and KEGG enrichment results suggested that the CNVs might contribute to various biological processes related to growth (regulation of actin cytoskeleton, and cAMP signaling pathway), lipid metabolism (phospholipid binding, sphingolipid metabolism, and regulation of lipolysis in adipocytes), behavior (axon guidance, circadian entrainment, and glutamatergic synapse), and immune response (Wnt signaling pathway, Fc receptor signaling pathway, and GTPase regulator activity). For instance, the most significantly enriched GO terms and KEGG results were related to axon guidance known as the key step in the formation of the neuronal network [80]. Interestingly, it was reported that CNVs might contribute to axonal growth, which has been connected with autism spectrum disorders [81]. The enrichment of several pathways related to lipid metabolism implied that CNVs might contribute to the fur growth and quality as fat metabolism is an important process during furring [82]. Circadian entrainment is an essential part of behavior and adaptation since it plays a fundamental role to assists organisms in adapting to daily environmental cycles [83]. Several studies demonstrated that the annual reproductive cycle in mink is under photoperiodic control, and is initiated by decreasing the daylength [84, 85]. It is well-documented that photoregulation of reproductive activity is associated with the circadian rhythm of photosensitivity, leading to a proper photoperiodic response in mink [86, 87]. Boissin-Agasse et al. [87] identified that seasonal testis activity in mink initiated in the Fall when the daily light period is decreasing and exposure to light at this period inhibited testicular development. Zschille et al. [88] reported different circadian activity rhythm in male and female mink, and observed active males during the night, and females with high activity during the day. Gender differences in circadian activity rhythms of wild American mink increases the female hunting successes as it allows females to be in a patch in different time than males to avoid the competitive pressure from the males [88]. In addition, several studies in mink have shown that decreasing the photoperiod in the Fall initiates winter fur growth and starting the hair growth in summer is associated with increasing photoperiod in spring [89–91]. Recently, Nandolo et al. [27] reported enrichment of circadian entrainment pathway among genes detected across the CNVs in African goats, supporting the importance of circadian entrainment in goats during the adaptation to unstable environment. Notably, it is well-documented that Wnt signaling pathway plays a key role in hair growth and development of hair follicles [92, 93]. The maintenance of Wnt signaling pathway is a critical part to hair-inducing activity of dermal papilla through regulating the β-catenin pathway, and thereby required for follicle regeneration and growth of the hair shaft [94, 95]. Interestingly, Yuan et al. [23] demonstrated the contribution of Wnt signaling pathway to the hair follicle development process in Alpine Merino sheep by identifying Wnt-related signaling pathways associated with CNVR-harboring genes [23].

In addition, GO enrichment and KEGG analyses identified several key genes (*MYO5A*, *RAB27B*, *FGF12*, *SLC7A11*, and *EXOC2*) participating in a wide range of pathways associated with fur characteristics and development. In this study, the *MYO5A* gene (CNVR_Chr13:75.88–75.89 Mb), a class of actin-based motor proteins, was enriched in several pathways such as actin filament organization, actin-based cell projection, calmodulin binding, actin binding, and cytoskeletal motor activity. The *MYO5A* gene is found in pigment-producing cells, which produce melanin and eventually provides the pigment required for normal color of hair, skin, and eye [96]. It has been suggested that *MYO5A* gene plays a key role in the industrial Silverblue coat color in American mink [97]. Several studies reported that the *MYO5A* gene can cause diluted (grey) coat color phenotype in different species, e.g., rabbit [98], horse [99], dog [100], and mice [101]. The *RAB27B,* which overlapped with CNVR_Chr3:143.66–143.67 Mb, is part of the small GTPase Ras-associated binding family that regulates the membrane trafficking and secretion of exosomes. It was indicated that *RAB27B* and its paralogue (the *RAB27A*), played some roles in the transport of melanosomes, and the knockout of this gene might cause silvery gray hair [102–104]. Recently, Ku et al. [105] reported that *RAB27A/B* played a regulating role for hair growth during the hair cycle in human. The *FGF12* gene overlapped with CNVR (Chr6:114.36–114.37 Mb), was related to hair growth development. Fibroblast growth factors (FGF) are a family of growth factors that are involved in the regulation of hair morphogenesis and cycle hair growth [106, 107]. Lv et al. [108] reported a regulating role of *FGF12* gene in the sheep hair follicle development process. In addition, our finding supported by Wang et al. [109] study that reported the role of *FGF12* gene in hair follicle development in cashmere goats. The *SLC7A11* gene (CNVR_Chr7:73.54–73.57 Mb) is an amino acid transporter which mediates the extracellular cysteine in exchange for glutamate [110]. It is well documented that the *SLC7A11* gene plays a critical role in changing the fur and skin color formation in animals through regulating the production of pheomelanin pigment [111–114]. The amino acid cysteine is necessary for the formation of disulfide bonds and crosslinking between cysteines in the keratins and hair keratin-associated proteins is proved to be as an important step in forming the fineness, length, flexibility and other physical properties of hair and wool fibers [115]. Thus, it was shown that the differences in the cysteine content leads to various structure of the hair fiber among species [116]. Cysteine is an integral part of the pheomelanin synthesis to construct yellow or red hair color in humans and animals as it regulates the conversion of dopaquinone to pheomelanin in hair follicle melanocytes [117, 118]. Chintala et al. [119] found that the subtle gray mouse pigmentation mutant is under the genetic control of a mutation form of *SLC7A11* gene as it affects the rate of extracellular cystine transport into melanocytes, which reduces pheomelanin production and consequently, the loss of yellow pigment. Moreover, Song et al. [120] identified the *SLC7A11* gene as one of the key genes associated with the development of black and white coat color in farmed mink. The *EXOC2* gene (CNVR_Chr1:123.59–123.60 Mb) has been previously found to be associated with pigmentary phenotypes such as hair color and skin pigmentation [121–123]. Our results suggested that these CNVR-harboring genes might be the potential candidate genes for fur characteristics and development in American mink.

Our results also revealed several CNVR-harbored genes related to the immune system process (*SWAP70*, *FYN*, *ORAI1*, *TRPM2,* and *FOXO3*). The *SWAP70* gene (CNVR_Chr11:157.8–157.9 Mb), is essential for normal B-cell migration that immobilizes F-actin filaments on phagosomes, contributing to immune regulation such as maturation and differentiation of immune cells [124, 125]. Interestingly, Karimi et al. [126] reported the *SWAP70* gene as a potential candidate gene for response to Aleutian mink disease virus infection. The *FYN* gene (CNVR_Chr1:20.84–20.85 Mb), which is involved in various signaling pathways, plays a critical role in apoptosis and immune response by regulating neuronal development and signaling in T and B cells [127, 128]. Zanella et al. [129] suggested the *FYN* gene as a functional candidate gene associating with immune response to vaccinated pigs against influenza virus. The *ORAI1* gene (CNVR_Chr3:234.70–234.71 Mb) was the other gene associated with immune response, which is an important signaling component required for T cell activation and function [130]. The *ORAI1* gene plays a role in maintaining a tick resistance status during the cattle tick infection [131]. Recently, Xue et al. [132] reported that the *ORAI1* might have regulating functions in the immune response, exacerbates inflammation and endoplasmic reticulum stress in bovine hepatocytes.

The *TRPM2* gene (CNVR_Chr6:1.82–1.83 Mb), which is a Ca^2+^-permeable cation channel, is highly expressed in immune cells, primarily polymorphonuclear leukocytes, monocytes/macrophages, and T-cells [133, 134]. It was revealed that *TRPM2*-deficient mice were highly susceptible to listeriosis infection, showing an ineffective innate immune response [135]. The *FOXO3* gene (CNVR_Chr1:23.49–23.50 Mb), which significantly enriched in Wnt signaling pathway, has been found to have therapeutic potential in chronic and autoimmune diseases [136]. Aleutian mink disease virus causes autoimmune disorders in mink, stimulating the immune responses to provide antibodies, and consequently forming the immune complexes [126, 137]. Taking into account that most of mink farms are challenged by Aleutian mink disease virus, the most prevalence disease in the worldwide mink industry, suggesting that these genes, and related pathways, might substantially contribute to the modulation of immune responses to Aleutian mink disease virus infection. Nevertheless, the above functional inference of CNVs is based on enrichment analyses of their annotated genes and mostly based on the results from studies in other species, therefore, further functional validation of these CNVs is required to confirm their functions in mink.

## Conclusions

In this study, we present the first CNV map of American mink using WGS data. We identified 5378 CNVR covering 1.9% of the mink autosome. Functional annotation revealed CNVR enriched for genes related to natural behavior, lipid metabolism, and immune response. Our results revealed several CNVR that harbor genes related to fur quality *(MYO5A*, *RAB27B*, *FGF12*, *SLC7A11*, and *EXOC2*), and immune system response (*SWAP70*, *FYN*, *ORAI1*, *TRPM2*, and *FOXO3*). Overall, the results of the current study may facilitate our further understanding of the genetic control of different characteristics of fur in American mink and immune responses to Aleutian mink disease virus infection, which is the most prevalence disease in the worldwide mink industry.

## Supplementary Information

**Additional file 1: Table S1.** List of CNVs identified on 100 American mink genomes. **Table S2.** Detail information of the detected CNVR. **Table S3.** List of genes completely/partially overlapped with CNVR in American mink. **Table S4.** Functional enrichment of gene ontology analysis of genes covered by CNVR. **Table S5.** Functional enrichment of KEGG pathway analysis of genes covered by CNVR.

## Data Availability

The datasets used and analyzed during the current study are available from the corresponding author on academic request.
